# The Routine Use of Prophylactic Oxytocin in the Third Stage of Labor to Reduce Maternal Blood Loss

**DOI:** 10.1155/2017/3274901

**Published:** 2017-09-11

**Authors:** Akiko Kuzume, So Sugimi, Sachie Suga, Hiroshi Yamashita, Ichiro Yasuhi

**Affiliations:** Department of Obstetrics and Gynecology, NHO Nagasaki Medical Center, Omura, Japan

## Abstract

**Objective:**

To demonstrate whether or not the routine use of prophylactic oxytocin (RUPO) reduces the blood loss and incidence of postpartum hemorrhaging (PPH).

**Methods:**

We used a prospective cohort and a historical control in a tertiary perinatal care center in Japan. In the prospective cohort, we introduced RUPO in April 2012 by infusing 10 units of oxytocin per 500 mL of normal saline into a venous line after anterior shoulder delivery (RUPO group). In the historical control, oxytocin was administered via a case-selective approach (historical control group). We included completed singleton vaginal deliveries and compared the volume of blood loss and the incidence of PPH between the groups.

**Results:**

We found a significantly lower volume of blood loss (520 ± 327 versus 641 ± 375 mL, *p* < 0.001) and a lower incidence of PPH (6.1% versus 14.0%, *p* < 0.001) in the RUPO group (*n* = 392) than in the control group (*n* = 407). Although the oxytocin dose was significantly higher in the RUPO group (12.8 ± 6.7 versus 10.1 ± 8.0 IU, *p* < 0.001), no adverse outcomes were observed to be associated with RUPO.

**Conclusions:**

The introduction of RUPO significantly reduced blood loss and the incidence of PPH during completed singleton vaginal deliveries without an increase in adverse effects.

## 1. Introduction

Postpartum hemorrhaging (PPH) is a major cause of maternal mortality in developing countries and is a significant cause of maternal morbidity in developed countries. Because PPH occurs suddenly in low-risk pregnancies, both prophylactic and therapeutic approaches are important for reducing blood loss and preventing PPH in all women at delivery.

The active management of the third stage of labor (AMTSL), which consists of the prophylactic administration of an uterotonic agent prior to placental separation, early cord clamping and traction, and uterine massage, is generally recommended. A recent Cochrane review [1] summarized the benefits and risks of AMTSL and concluded that although there is a lack of high-quality evidence, AMTSL reduced the risk of hemorrhaging > 1,000 mL at the time of birth in a population of women with a mixed risk of excessive bleeding. Regarding cord traction [2–4] and uterine massage [5, 6], the effects of reducing maternal blood loss have been controversial. In addition, the most common cause of PPH is uterine atony, which complicates 1 in 20 deliveries and is responsible for 80% of PPH cases [7]. Thus, the administration of a uterotonic agent seems to be an essential component of AMTSL to prevent PPH.

Although AMTSL is also recommended and oxytocin is the most widely used uterotonic agent in Japan [8], there have been few studies regarding the prophylactic effects of either the administration of oxytocin or AMTSL in the prevention of PPH in the Japanese population. Furthermore, a recent systematic review [1] reported that the benefits of AMTSL were less robust in women who were at low risk of PPH. Therefore, whether or not the routine use of prophylactic oxytocin (RUPO) more efficiently reduces blood loss and is protective against PPH in the third stage of labor compared with the selective use of oxytocin in women with risk factors for PPH remains unclear.

In the present study, we aimed to demonstrate whether or not RUPO, as a component of AMTSL, effectively reduces maternal blood loss and PPH compared with the physician-oriented selective use of oxytocin.

## 2. Materials and Methods

In the present study we used a prospective cohort and a historical control in a single tertiary perinatal care center to investigate the impact of RUPO as a component of AMTSL. On April 1, 2012, we introduced RUPO for all women who delivered vaginally in our delivery units. Our RUPO protocol involved the infusion of 10 units of oxytocin with 500 mL of 0.9% normal saline (NS) into a maternal venous line at a rate of 250 to 500 mL/h just after the delivery of the infant's anterior shoulder. When the infusion was completed, a second infusion of 10 units of oxytocin with the same volume of NS was allowed to be administered, depending on the situation. In the era before the introduction of RUPO, we did not routinely administer oxytocin or perform other AMTSL procedures; instead, we adopted a case-selective approach in which these measures could be implemented at any time after placental delivery, depending on the attending physicians.

Consecutive cases of completed singleton vaginal deliveries between April 1, 2012, and March 31, 2013, were used as a prospective cohort (RUPO group), while consecutive cases of completed singleton vaginal deliveries between April 1, 2011, and March 31, 2012, were used as a historical control (control group). We excluded cases with breech presentation and women who delivered at <24 weeks of gestation. While AMTSL procedures, including cord traction and uterine massage, were routinely performed in the RUPO group, the choice to implement such measures in the control group was left to the decision of the attending physicians. Early cord clamping was not used routinely in either group. The clinical data in each group were obtained by a chart review.

The primary outcomes of this study were the volume of maternal blood loss during the first 2 h after delivery and the prevalence of PPH ≥ 1,000 mL during the first 24 h after birth. We compared the main outcomes between the groups.

We also compared the prevalence of PPH ≥ 1,500 mL, the dose of oxytocin, the use of uterotonic agents in addition to oxytocin, the duration of the third stage of labor, the incidence of the manual removal of the placenta, and other complications between groups. Prolonged labor was defined as more than 15 and 30 h, and a prolonged second stage of labor was defined as 1.5 and 3 h in multiparous and nulliparous women, respectively, according to the definitions of the Japan Society of Obstetrics and Gynecology.

Student's *t*-test and the chi-squared test were used to compare differences between the groups. *p* values of <0.05 were considered to indicate statistical significance. This study was conducted with the approval of the Institution Review Board of Nagasaki Medical Center. The patients provided their informed consent for the collection of their clinical data according to our clinical data utilization policy for the purpose of clinical research established in 2008.

## 3. Results

The RUPO and control groups included 392 and 407 women, respectively. Although the patient characteristics and the perinatal outcomes did not differ markedly between the groups, the women in the RUPO group showed significantly higher prepregnancy body mass index (BMI) values and a higher prevalence of prolonged labor than the control subjects (Table 1).

The rate of oxytocin use was 71% in the control group, and the dose of oxytocin was significantly higher in the RUPO group (Table 2). The rates at which additional uterotonic agents (besides oxytocin) were used did not differ to a statistically significant extent. With regard to the main outcomes, we found that the women in the RUPO group showed a significantly smaller volume of blood loss than those in the control group and less than half the incidence of PPH ≥ 1,000 mL. The incidence of PPH ≥ 1,500 mL in the RUPO group was also approximately half of that in the control group; however, the result was not statistically significant (Table 2). The rate at which the placenta was manually removed did not differ markedly between the groups. No other adverse outcomes, such as RUPO practice-induced hypertension or after-pains requiring analgesia, were observed in the RUPO group.

## 4. Discussion

We found that the volume of maternal blood loss in the RUPO group was reduced by 20% compared with the historical control group. The incidence of PPH ≥ 1,000 mL was also significantly reduced from 14.0% in the control group to 6.1% in the RUPO group, despite the women in the RUPO group being more obese and having a longer first stage of labor, both of which are risk factors of excessive bleeding. The incidence of severe PPH (≥1500 mL) was also half of that in the control group (1.5% versus 3.2%); however, it did not reach statistical significance. Thus, our results showed that RUPO, as a component of AMTSL, contributed to the reduction of both maternal blood loss and PPH at the time of delivery. The routine administration of oxytocin in all cases of vaginal delivery seems to be superior in the prevention of PPH to the physician-dependent administration of oxytocin in select cases.

Uterine atony is responsible for more than 80% of PPH cases [7]; the administration of uterotonic agents is therefore a logical and essential component of AMTSL to prevent PPH. Oxytocin is generally the initial drug of choice for preventing PPH. A Cochrane systematic review in 2013 [9] demonstrated that prophylactic use of oxytocin significantly reduced the risk of blood loss of >500 mL due to PPH (average risk ratio [RR] 0.53; 95% confidence interval [CI] 0.38 to 0.74) in comparison to a placebo. Nevertheless, some authors still consider the suggestion that the routine application of AMTSL is superior to expectant management in all vaginal deliveries, including women with a low risk of excessive bleeding, to be controversial, due to the possibility of adverse effects in association with AMTSL, especially the potential for overdose when uterotonic agents are administered [1]. Although the mean dose of oxytocin in the RUPO group was significantly higher than that in the control group, the mean difference in the oxytocin dose was only 2.7 IU (Table 2), which does not seem to be harmful. In addition, no adverse outcomes were observed in the RUPO group.

The present study is associated with some limitations. Our study was not a randomized control trial, and a retrospective historical cohort was used for the control group. We were therefore not able to obtain detailed data, especially in relation to the AMTSL procedures that were performed in the historical control group. Thus, it was unclear whether oxytocin was administered for prophylactic or therapeutic purposes in the control group; furthermore, the cases in which AMTSL was practiced were unclear. Thus, the difference in the AMTSL practices of the groups may have affected the results. However, because the procedures, including cord traction and uterine massage, are considered to have little or no effect [3, 4, 6], we believe that RUPO reduced the maternal blood loss.

In addition, both the administration of oxytocin and the timing of the administration might have led to reduced blood loss. In the RUPO group, the administration of oxytocin was started just after the delivery of the anterior fetal shoulder, which was earlier than in the control group, in which oxytocin infusion began after placental delivery. A recent systematic review [9] suggested that giving uterotonic therapy before the delivery of the placenta results in a lower volume of blood loss and fewer postpartum transfusions.

## 5. Conclusions

Our results showed that the routine prophylactic use of oxytocin as a component of AMTSL efficiently reduced the volume of blood loss and the incidence of PPH without a significant increase in the incidence of adverse outcomes.

## Figures and Tables

**Table 1:** The maternal characteristics and perinatal outcomes in each group.

	Control group (*n* = 407)	RUPO group (*n* = 392)	*p* value
Maternal age (years)	30.9 ± 5.5	30.9 ± 5.5	NS
Nulliparous (%)	221 (54.3%)	196 (50.0%)	NS
Prepregnancy BMI (kg/m^2^)	21.0 ± 3.9	21.5 ± 4.9	NS
Prepregnancy obesity (BMI ≥ 25)	41 (10.1%)	61 (15.5%)	*p* < 0.05
Leiomyomas (%)	10 (2.5%)	12 (3.1%)	NS
Preeclampsia (%)	11 (2.7%)	8 (2.0%)	NS
Hydramnios (%)	14 (3.4%)	10 (2.5%)	NS
Gestational age at delivery (weeks)	38.4 ± 2.8	38.3 ± 2.6	NS
Birthweight (g)	2,895 ± 619	2,871 ± 561	NS
Macrosomia ≥ 4,000 g (%)	6 (1.5%)	3 (0.8%)	NS
Induction of labor with oxytocin (%)	106 (26%)	107 (27%)	NS
Duration of labor (min)	493 ± 396	482 ± 394	NS
Prolonged labor (%)	12 (2.9%)	33 (8.4%)	*p* < 0.001
Duration of the second stage of labor (min)	67.3 ± 111	59.9 ± 94	NS
Prolonged second stage of labor (%)	73 (17.9%)	70 (17.8%)	NS
Vacuum extraction (%)	20 (4.9%)	20 (5.1%)	NS
Cervical laceration (%)	3 (0.7%)	0 (0%)	NS

RUPO, routine use of prophylactic oxytocin; BMI, body mass index.

**Table 2:** The main outcomes and other factors associated with RUPO.

	Control group (*n* = 407)	RUPO group (*n* = 392)	*p* value
Maternal blood loss (ml)	641 ± 373	520 ± 327	*p* < 0.001
Median (range) (ml)	562 (96–3,404)	428 (68–2,860)	
PPH ≥ 1,000 mL (%)	57 (14.0%)	24 (6.1%)	*p* < 0.001
PPH ≥ 1,500 mL (%)	13 (3.2%)	6 (1.5%)	NS (*p* = 0.12)
Blood transfusion (%)	3 (0.7%)	2 (0.5%)	NS
Rate of oxytocin use (%)	289 (71%)	392 (100%)	
Dose of oxytocin (IU)	10.1 ± 8.0	12.8 ± 6.7	*p* < 0.001
Additional uterotonic agents (%)	27 (6.6%)	40 (10.2%)	NS
Duration of third stage of labor (min)	5.8 ± 6.4	6.1 ± 15.1	NS
Manual removal of placenta (%)	7 (1.7%)	7 (1.8%)	NS

RUPO, routine use of prophylactic oxytocin; PPH, postpartum hemorrhage.
